# Supplementary material for: Does Better Diet Quality Offset the Association between Depression and Metabolic Syndrome?
Source: Nutrients. 2023 Feb 20;15(4):1060. doi: 10.3390/nu15041060 (PMC9962849; doi:10.3390/nu15041060)
Supplement: Supplementary file 1 [file nutrients-15-01060-s001.zip › nutrients-2214360-supplementary.pdf]

Supplementary Table S1. Mediating effects of diet quality on the association between depression severity and metabolic syndrome

	Metabolic syndrome		
	**Model 1**<br>**X -> Y**	**Model 2**<br>**X + M -> Y**	**Proportion of mediation**
Depression severity<br>(Independent Variable, X)	1.47<br>(1.17-1.86) **	1.02<br>(1.00-1.03) *	
Adequacy item<br>(Mediator, M)		0.87<br>(0.75 – 1.00)	
Depression severity<br>(Independent Variable, X)	1.47<br>(1.17-1.86) **	1.47<br>(1.17-1.87) **	Fail to meet the conditions
Moderation item<br>(Mediator, M)		1.12<br>(0.96 – 1.32)	
Depression severity<br>(Independent Variable, X)	1.47<br>(1.17-1.86) **	1.48<br>(1.17-1.87) **	
Balance item<br>(Mediator, M)		1.06<br>(0.93-1.21)	

Values are presented as adjusted odds ratios (95% confidence intervals) adjusted for age, sex, body mass index, education level, drinking consumption, energy intake, and physical activity. *$p < 0.05$. **$p < 0.01$.

Supplementary Table S2. Mediating effects of fulfilling subcomponents of Korean Healthy Eating Index on the association between depression severity and metabolic syndrome

	Metabolic syndrome (Dependent variable, Y)		
	**Model 1 X -> Y**	**Model 2 X + M -> Y**	**Proportion of mediation**
Depression severity (Independent Variable, X)	1.47 (1.17-1.86) **	1.46 (1.16-1.85) **	
Have breakfast (Mediator, M)		0.89 (0.78-1.01)	Fail to meet the conditions
Depression severity (Independent Variable, X)	1.47 (1.17-1.86) **	1.47 (1.17-1.86) **	
Mixed grain intake (Mediator, M)		0.92 (0.81-1.04)	
Depression severity (Independent Variable, X)	1.47 (1.17-1.86) **	1.47 (1.16-1.86) **	Fail to meet the conditions (because of X->M: p=0.172)
Total fruits intake (Mediator, M)		0.79 (0.71-0.90) **	
Depression severity (Independent Variable, X)	1.47 (1.17-1.86) **	1.47 (1.16-1.86) **	Fail to meet the conditions (because of X->M: p=0.247)
Fresh fruits intake (Mediator, M)		0.81 (0.73-0.91) **	
Depression severity (Independent Variable, X)	1.47 (1.17-1.86) **	1.48 (1.17-1.87) **	63.3%
Total vegetables intake (Mediator, M)		1.13 (1.01-1.27) *	
Depression severity (Independent Variable, X)	1.47 (1.17-1.86) **	1.48 (1.17-1.87) **	66.3%
Vegetable intake excluding kimchi and pickled vegetables (Mediator, M)		1.14 (1.01-1.28) *	
Depression severity (Independent Variable, X)	1.47 (1.17-1.86) **	1.47 (1.17-1.86) **	
Meats/fishes/eggs/beans intake (Mediator, M)		0.99 (0.88-1.11)	
Depression severity (Independent Variable, X)	1.47 (1.17-1.86) **	1.44 (1.11-1.86)	
Milk and dairy product intake (Mediator, M)		0.91 (0.81-1.03)	
Depression severity (Independent Variable, X)	1.47 (1.17-1.86) **	1.47 (1.17-1.86) **	Fail to meet the conditions
Energy from saturated fatty acid (Mediator, M)		1.07 (0.95-1.20)	
Depression severity (Independent Variable, X)	1.47 (1.17-1.86) **	1.46 (1.16-1.85) **	
Sodium intake (Mediator, M)		1.09 (0.95-1.26)	
Depression severity (Independent Variable, X)	1.47 (1.17-1.86) **	1.48 (1.17-1.87) **	
Energy from sweets/beverages (Mediator, M)		1.11 (0.97-1.27)	

Depression severity<br>(Independent Variable, X)	1.47<br>(1.17-1.86) **	1.47<br>(1.17-1.86) **	
Energy from carbohydrate<br>(Mediator, M)		1.02<br>(0.91-1.15)	
Depression severity<br>(Independent Variable, X)	1.47<br>(1.17-1.86) **	1.47<br>(1.17-1.86) **	
Energy from fat<br>(Mediator, M)		0.96<br>(0.87-1.07)	
Depression severity<br>(Independent Variable, X)	1.47<br>(1.17-1.86) **	1.47<br>(1.16-1.86) **	
Total energy intake<br>(Mediator, M)		1.00<br>(0.89-1.11)	

KHEI: Korean Healthy Eating Index. Values are presented as adjusted odds ratios (95% confidence intervals) adjusted for age, sex, body mass index, education level, drinking consumption, energy intake, and physical activity. *$p < 0.05$. **$p < 0.01$

Supplementary Table S3. Scores of Korean healthy eating index of subjects according to fulfilling both vegetable intake subcomponents

	**Full scores of both vegetable intake subcomponents**		
	**No (N =10,353)**	**Yes (N=3186)**	***p*-value**
Total score	61.90±0.17	66.53±0.26	<0.001
Adequacy item			
Have breakfast	7.00±0.05	7.61±0.08	<0.001
Mixed grain intake	1.96±0.03	2.33±0.05	<0.001
Total fruits intake	2.19±0.03	2.26±0.04	0.156
Fresh fruits intake	2.39±0.03	2.43±0.05	0.371
Total vegetables intake	3.13±0.02	Full score (5)	<0.001
Vegetable intake excluding kimchi and pickled vegetables	2.78±0.02	Full score (5)	<0.001
Meats/fishes/eggs/beans intake	7.03±0.04	7.60±0.05	<0.001
Milk and dairy product intake	3.43±0.06	2.97±0.10	<0.001
Moderation item			
Energy from saturated fatty acid	7.26±0.05	8.11±0.08	<0.001
Sodium intake	6.82±0.03	5.37±0.06	<0.001
Energy from sweets/beverages	9.07±0.03	9.30±0.04	<0.001
Balance item			
Energy from carbohydrate	2.46±0.02	2.64±0.05	0.001
Energy from fat	3.29±0.02	3.45±0.04	0.002
Total energy intake	3.09±0.03	2.95±0.05	<0.001

Values are presented as mean ± standard error. [a-c] Significantly different at $p < 0.05$. Adjusted for age, sex, body mass index, education level, alcohol drinking consumption, energy intake, disease history, and physical activity.

Supplementary Table S4. Nutrient intakes of subjects according to fulfilling both vegetable intake subcomponents

	Full scores of both vegetable intake subcomponents		
	**No (N =10,353)**	**Yes (N=3186)**	***p*-value**
Protein, g	72.16±0.31	80.79±0.63	<0.001
Fat, g	47.56±0.30	47.51±0.59	0.948
Saturated fatty acid, g	15.20±0.13	13.42±0.21	<0.001
Monounsaturated fatty acid, g	15.26±0.12	15.26±0.25	0.994
Polyunsaturated fatty acid, g	11.79±0.09	13.49±0.20	<0.001
n-3 fatty acid, g	1.72±0.02	2.22±0.04	<0.001
n-6 fatty acid, g	10.07±0.08	11.30±0.17	<0.001
Cholesterol, mg	254.14±2.59	270.14±5.19	0.008
Carbohydrate, g	303.42±1.08	315.93±1.87	<0.001
Total fiber, g	23.10±0.15	31.93±0.28	<0.001
Calcium, mg	489.43±3.53	609.24±8.03	<0.001
Phosphate, mg	1059.75±3.64	1249.42±7.85	<0.001
Iron, mg	13.11±0.19	17.19±0.28	<0.001
Sodium, mg	3436.02±22.38	4413.68±51.64	<0.001
Potassium, mg	2747.78±12.47	3680.09±27.27	<0.001
Carotene, μg	2514.64±52.24	5331.41±133.37	<0.001
Retinol, μg	151.51±5.90	139.69±17.14	0.543
Thiamin, mg	1.50±0.01	1.91±0.02	<0.001
Riboflavin, mg	1.15±0.01	1.78±0.02	<0.001
Niacin, mg	14.28±0.07	17.02±0.17	<0.001
Vitamin C, mg	66.30±1.25	105.67±2.20	<0.001

Values are presented as mean ± standard error. [a-c] Significantly different at $p < 0.05$. Adjusted for age, sex, body mass index, education level, alcohol drinking consumption, energy intake, disease history, and physical activity.

Supplementary Table S5. Adjusted odds ratios (95% confidence intervals) for risks of metabolic syndrome and its components of subjects according to depression severity after stratified by full scores of both vegetable intake subcomponents measured by Korean Healthy Eating Index

	Full scores of both vegetable intake subcomponents							
	No (N =10,353)				Yes (N=3186)			
	PHQ-9 depression severity				PHQ-9 depression severity			
	Normal (N=8233)	Mild (N = 1501)	Moderate to severe (N = 619)	*p* trend	Normal (N=2655)	Mild (N = 404)	Moderate to severe (N = 127)	*p* trend
Metabolic syndrome	1 (Reference)	1,05 (0.88 – 1.25)	1.49 (1.14 – 1.94) **	0.007	1 (Reference)	0.89 (0.66 – 1.20)	1.45 (0.89 – 2.35)	0.573
Abdominal obesity[1]		1.19 (0.92 – 1.53)	1.06 (0.74 – 1.51) *	0.301		0.95 (0.60 – 1.49)	1.53 (0.74 – 3.18)	0.492
Hypertriglyceridemia[2]		1.15 (0.99 – 1.33) *	1.32 (1.05 – 1.64) **	0.005		1.11 (0.85 – 1.45) *	1.26 (0.75 – 2.10)	0.258
Low HDL cholesterol[3]		1.23 (1.07 – 1.42) **	1.30 (1.04 – 1.63) *	<0.001		1.18 (0.89 – 1.57)	0.91 (0.59 – 1.40)	0.585
High blood pressure[4]		0.87 (0.74 – 1.02)	0.92 (0.72 – 1.17)	0.138		0.76 (0.80 – 1.12)	0.80 (0.51 – 1.24)	0.047
Hyperglycemia[5]		0.96 (0.83 – 1.11)	1.22 (0.99 – 1.51)	0.242		0.93 (0.71 – 1.22)	1.30 (0.78 – 2.18)	0.647

PHQ-9: Patient Health Questionnaire-9. PHQ-9 depression severity was divided by total scores of PHQ-9 (normal: <5, mild: 5-9, moderate to severe: ≥10). KHEI: Korean Healthy Eating Index. Diet quality level was divided into tertiles by total scores of KHEI (Low (T1): < 58, Medium (T2): 58-69, High (T3): ≥70). [1] Waist circumference ≥ 90cm for men, ≥ 80cm for women. [2] Serum triglyceride level ≥ 150 mg/dL or current drug treatment for high triglyceride. [3] HDL-cholesterol < 40mg/dL for men, 50mg/dL for women. [4] Systolic blood pressure > 130 mmHg or diastolic blood pressure > 85 mmHg or current drug treatment for hypertension. [5] Fasting blood glucose ≥ 100 mg/dL or current drug treatment for hypoglycemic agent or insulin. Values are presented as adjusted odds ratios (95% confidence intervals) adjusted for age, sex, body mass index, education level, drinking consumption, energy intake, and physical activity. *$p < 0.05$, **$p < 0.01$.
